# Methyl 3-(4-methyl­benzyl­idene)carbazate

**DOI:** 10.1107/S160053681001799X

**Published:** 2010-05-22

**Authors:** Yu-Feng Li, Fu-Gong Zhang, Fang-Fang Jian

**Affiliations:** a Microscale Science Institute, Department of Chemistry and Chemical Engineering, Weifang University, Weifang 261061, People’s Republic of China; b Ministry of Personnel, Weifang University, Weifang 261061, People’s Republic of China; c Microscale Science Institute, Weifang University, Weifang 261061, People’s Republic of China

## Abstract

The title compound, C_10_H_12_N_2_O_2_, was prepared by the reaction of methyl carbazate and 4-methyl­benzaldehyde. The dihedral angle between the benzene ring and the carbazate fragment is 20.86 (10)°. In the crystal structure, mol­ecules are linked by inter­molecular N—H⋯O hydrogen bonds.

## Related literature

For background to Schiff bases, see: Cimerman *et al.* (1997 ▶). For C=N bond lengths, see: Girgis (2006 ▶). For a related structure, see: Li *et al.* (2009 ▶).
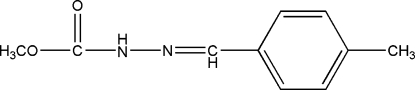


## Experimental

### 

#### Crystal data

C_10_H_12_N_2_O_2_
*M* *_r_* = 192.22
Monoclinic, 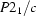

*a* = 10.038 (2) Å
*b* = 13.308 (3) Å
*c* = 7.7923 (16) Å
β = 99.71 (3)°
*V* = 1026.1 (4) Å^3^
*Z* = 4
Mo *K*α radiation
μ = 0.09 mm^−1^
*T* = 293 K
0.22 × 0.20 × 0.18 mm

#### Data collection

Bruker SMART CCD area-detector diffractometer
9493 measured reflections
2322 independent reflections
1528 reflections with *I* > 2σ(*I*)
*R*_int_ = 0.043

#### Refinement

*R*[*F* ^2^ > 2σ(*F* ^2^)] = 0.048
*wR*(*F* ^2^) = 0.175
*S* = 1.06
2322 reflections
127 parameters
H-atom parameters constrained
Δρ_max_ = 0.26 e Å^−3^
Δρ_min_ = −0.23 e Å^−3^

### 

Data collection: *SMART* (Bruker, 1997 ▶); cell refinement: *SAINT* (Bruker, 1997 ▶); data reduction: *SAINT*; program(s) used to solve structure: *SHELXS97* (Sheldrick, 2008 ▶); program(s) used to refine structure: *SHELXL97* (Sheldrick, 2008 ▶); molecular graphics: *SHELXTL* (Sheldrick, 2008 ▶); software used to prepare material for publication: *SHELXTL*.

## Supplementary Material

Crystal structure: contains datablocks global, I. DOI: 10.1107/S160053681001799X/hg2684sup1.cif

Structure factors: contains datablocks I. DOI: 10.1107/S160053681001799X/hg2684Isup2.hkl

Additional supplementary materials:  crystallographic information; 3D view; checkCIF report

## Figures and Tables

**Table 1:** Hydrogen-bond geometry (Å, °)

*D*—H⋯*A*	*D*—H	H⋯*A*	*D*⋯*A*	*D*—H⋯*A*
N1—H1*A*⋯O1^i^	0.86	2.00	2.8615 (18)	176

Symmetry code: (i) 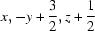.

**Fig. 1.**
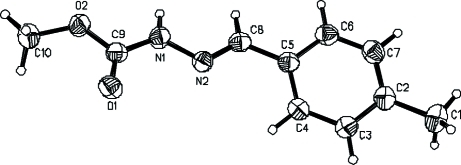
The structure of the title compound showing 30% probability displacement ellipsoids and the atom-numbering scheme.

## supplementary crystallographic information

### Comment

Schiff bases have received considerable attention in the literature. They are attractive from several points of view, such as the possibility of analytical application (Cimerman, *et al.*, 1997). As part of our search for new Schiff base compounds we synthesized the title compound (I), and describe its structure here.

The molcular structure of (I) is shown in Fig. 1. The C8—N2 bond length of 1.273 (2)Å is comparable with C—N double bond [1.281 (2) Å] reported (Girgis, 2006). In the crystal structure, molecules are linked by intermolecular N—H···O hydrogen bonds.

### Experimental

A mixture of methyl carbazate (0.1 mol), and 4-methylbenzaldehyde (0.1 mol) was stirred in refluxing ethanol (20 mL) for 4 h to afford the title compound (0.085 mol, yield 85%). Single crystals suitable for X-ray measurements were obtained by recrystallization from ethanol at room temperature.

### Refinement

H atoms were fixed geometrically and allowed to ride on their attached atoms, with C—H distances = 0.93-0.97 Å; N—H = 0.86Å and with *U*_iso_(H) = 1.2*U*_eq_(C,*N*) or 1.5*U*_eq_(C_methyl_).

### Crystal data

**Table:**

C_10_H_12_N_2_O_2_	*F*(000) = 408
*M**_r_* = 192.22	*D*_x_ = 1.244 Mg m^−^^3^
Monoclinic, *P*2_1_/*c*	Mo *K*α radiation, λ = 0.71073 Å
Hall symbol: -P 2ybc	Cell parameters from 1798 reflections
*a* = 10.038 (2) Å	θ = 3.5–25.2°
*b* = 13.308 (3) Å	µ = 0.09 mm^−^^1^
*c* = 7.7923 (16) Å	*T* = 293 K
β = 99.71 (3)°	Blcok, colorless
*V* = 1026.1 (4) Å^3^	0.22 × 0.20 × 0.18 mm
*Z* = 4	

### Data collection

**Table:**

Bruker SMART CCD area-detector diffractometer	1528 reflections with *I* > 2σ(*I*)
Radiation source: fine-focus sealed tube	*R*_int_ = 0.043
graphite	θ_max_ = 27.5°, θ_min_ = 3.1°
phi and ω scans	*h* = −13→12
9493 measured reflections	*k* = −17→17
2322 independent reflections	*l* = −9→10

### Refinement

**Table:**

Refinement on *F*^2^	Primary atom site location: structure-invariant direct methods
Least-squares matrix: full	Secondary atom site location: difference Fourier map
*R*[*F*^2^ > 2σ(*F*^2^)] = 0.048	Hydrogen site location: inferred from neighbouring sites
*wR*(*F*^2^) = 0.175	H-atom parameters constrained
*S* = 1.06	*w* = 1/[σ^2^(*F*_o_^2^) + (0.1077*P*)^2^] where *P* = (*F*_o_^2^ + 2*F*_c_^2^)/3
2322 reflections	(Δ/σ)_max_ < 0.001
127 parameters	Δρ_max_ = 0.26 e Å^−^^3^
0 restraints	Δρ_min_ = −0.23 e Å^−^^3^

### Special details

**Table:**

Geometry. All esds (except the esd in the dihedral angle between two l.s. planes) are estimated using the full covariance matrix. The cell esds are taken into account individually in the estimation of esds in distances, angles and torsion angles; correlations between esds in cell parameters are only used when they are defined by crystal symmetry. An approximate (isotropic) treatment of cell esds is used for estimating esds involving l.s. planes.
Refinement. Refinement of *F*^2^ against ALL reflections. The weighted *R*-factor wR and goodness of fit *S* are based on *F*^2^, conventional *R*-factors *R* are based on *F*, with *F* set to zero for negative *F*^2^. The threshold expression of *F*^2^ > σ(*F*^2^) is used only for calculating *R*-factors(gt) etc. and is not relevant to the choice of reflections for refinement. *R*-factors based on *F*^2^ are statistically about twice as large as those based on *F*, and *R*- factors based on ALL data will be even larger.

### Fractional atomic coordinates and isotropic or equivalent isotropic displacement parameters (Å^2^)

**Table:**

	*x*	*y*	*z*	*U*_iso_*/*U*_eq_	
O2	0.44060 (12)	0.82548 (9)	0.19195 (16)	0.0656 (4)	
C9	0.55062 (16)	0.81674 (12)	0.1159 (2)	0.0531 (4)	
N2	0.75692 (13)	0.73089 (10)	0.14969 (17)	0.0559 (4)	
O1	0.56744 (12)	0.86340 (9)	−0.01208 (15)	0.0636 (4)	
C5	0.95735 (16)	0.62998 (12)	0.1941 (2)	0.0540 (4)	
N1	0.63541 (14)	0.74964 (11)	0.20247 (18)	0.0617 (4)	
H1A	0.6141	0.7184	0.2906	0.074*	
C8	0.82561 (17)	0.66004 (13)	0.2311 (2)	0.0580 (4)	
H8A	0.7903	0.6261	0.3176	0.070*	
C3	1.15344 (17)	0.65532 (14)	0.0599 (2)	0.0661 (5)	
H3A	1.2004	0.6951	−0.0078	0.079*	
C2	1.20961 (17)	0.56483 (14)	0.1252 (2)	0.0623 (5)	
C7	1.13692 (18)	0.50763 (15)	0.2245 (2)	0.0683 (5)	
H7A	1.1719	0.4465	0.2692	0.082*	
C6	1.01357 (17)	0.53927 (13)	0.2585 (2)	0.0629 (5)	
H6A	0.9668	0.4992	0.3260	0.076*	
C4	1.02987 (18)	0.68754 (13)	0.0931 (2)	0.0647 (5)	
H4A	0.9947	0.7484	0.0475	0.078*	
C10	0.3380 (2)	0.89244 (15)	0.1047 (3)	0.0789 (6)	
H10A	0.2630	0.8938	0.1665	0.118*	
H10B	0.3080	0.8693	−0.0120	0.118*	
H10C	0.3749	0.9589	0.1017	0.118*	
C1	1.3469 (2)	0.53239 (18)	0.0914 (3)	0.0857 (6)	
H1B	1.3699	0.4687	0.1463	0.128*	
H1C	1.4131	0.5815	0.1383	0.128*	
H1D	1.3449	0.5263	−0.0317	0.128*	

### Atomic displacement parameters (Å^2^)

**Table:**

	*U*^11^	*U*^22^	*U*^33^	*U*^12^	*U*^13^	*U*^23^
O2	0.0612 (7)	0.0707 (8)	0.0700 (8)	0.0078 (5)	0.0254 (6)	0.0082 (6)
C9	0.0607 (10)	0.0489 (8)	0.0527 (9)	−0.0029 (7)	0.0181 (8)	−0.0056 (7)
N2	0.0573 (8)	0.0566 (8)	0.0561 (8)	0.0004 (6)	0.0165 (6)	−0.0012 (6)
O1	0.0813 (9)	0.0577 (7)	0.0566 (7)	0.0059 (6)	0.0251 (6)	0.0061 (5)
C5	0.0561 (9)	0.0530 (9)	0.0517 (9)	−0.0046 (7)	0.0059 (7)	−0.0048 (7)
N1	0.0632 (9)	0.0686 (9)	0.0580 (9)	0.0076 (7)	0.0241 (7)	0.0119 (7)
C8	0.0613 (10)	0.0600 (10)	0.0535 (9)	−0.0063 (8)	0.0121 (8)	0.0016 (7)
C3	0.0633 (10)	0.0656 (11)	0.0719 (12)	−0.0027 (8)	0.0187 (9)	0.0010 (8)
C2	0.0575 (9)	0.0670 (11)	0.0597 (10)	0.0026 (8)	0.0025 (8)	−0.0105 (8)
C7	0.0672 (11)	0.0635 (11)	0.0698 (11)	0.0086 (8)	−0.0011 (9)	0.0068 (8)
C6	0.0629 (10)	0.0635 (10)	0.0606 (10)	−0.0035 (8)	0.0052 (8)	0.0096 (8)
C4	0.0675 (11)	0.0547 (9)	0.0736 (12)	0.0034 (8)	0.0169 (9)	0.0050 (8)
C10	0.0678 (12)	0.0721 (12)	0.0987 (15)	0.0134 (9)	0.0196 (11)	0.0115 (11)
C1	0.0677 (12)	0.1015 (15)	0.0883 (14)	0.0137 (11)	0.0145 (10)	−0.0081 (12)

### Geometric parameters (Å, °)

**Table:**

O2—C9	1.343 (2)	C3—H3A	0.9300
O2—C10	1.442 (2)	C2—C7	1.379 (3)
C9—O1	1.2108 (19)	C2—C1	1.509 (3)
C9—N1	1.335 (2)	C7—C6	1.375 (2)
N2—C8	1.273 (2)	C7—H7A	0.9300
N2—N1	1.3742 (19)	C6—H6A	0.9300
C5—C4	1.389 (2)	C4—H4A	0.9300
C5—C6	1.390 (2)	C10—H10A	0.9600
C5—C8	1.456 (2)	C10—H10B	0.9600
N1—H1A	0.8600	C10—H10C	0.9600
C8—H8A	0.9300	C1—H1B	0.9600
C3—C4	1.378 (2)	C1—H1C	0.9600
C3—C2	1.389 (3)	C1—H1D	0.9600
			
C9—O2—C10	114.86 (13)	C6—C7—H7A	119.4
O1—C9—N1	126.40 (15)	C2—C7—H7A	119.4
O1—C9—O2	123.93 (15)	C7—C6—C5	121.34 (17)
N1—C9—O2	109.67 (14)	C7—C6—H6A	119.3
C8—N2—N1	114.77 (14)	C5—C6—H6A	119.3
C4—C5—C6	117.63 (16)	C3—C4—C5	120.62 (17)
C4—C5—C8	122.74 (16)	C3—C4—H4A	119.7
C6—C5—C8	119.61 (16)	C5—C4—H4A	119.7
C9—N1—N2	119.52 (13)	O2—C10—H10A	109.5
C9—N1—H1A	120.2	O2—C10—H10B	109.5
N2—N1—H1A	120.2	H10A—C10—H10B	109.5
N2—C8—C5	122.59 (16)	O2—C10—H10C	109.5
N2—C8—H8A	118.7	H10A—C10—H10C	109.5
C5—C8—H8A	118.7	H10B—C10—H10C	109.5
C4—C3—C2	121.59 (17)	C2—C1—H1B	109.5
C4—C3—H3A	119.2	C2—C1—H1C	109.5
C2—C3—H3A	119.2	H1B—C1—H1C	109.5
C7—C2—C3	117.61 (17)	C2—C1—H1D	109.5
C7—C2—C1	121.67 (17)	H1B—C1—H1D	109.5
C3—C2—C1	120.70 (18)	H1C—C1—H1D	109.5
C6—C7—C2	121.21 (17)		

### Hydrogen-bond geometry (Å, °)

**Table:**

*D*—H···*A*	*D*—H	H···*A*	*D*···*A*	*D*—H···*A*
N1—H1A···O1^i^	0.86	2.00	2.8615 (18)	176

Symmetry codes: (i) *x*, −*y*+3/2, *z*+1/2.
